# Structural Basis of the Selective Block of Kv1.2 by Maurotoxin from Computer Simulations

**DOI:** 10.1371/journal.pone.0047253

**Published:** 2012-10-10

**Authors:** Rong Chen, Shin-Ho Chung

**Affiliations:** Research School of Biology, Australian National University, Canberra, Australian Capital Territory, Australia; Virginia Commonwealth University, United States of America

## Abstract

The 34-residue polypeptide maurotoxin (MTx) isolated from scorpion venoms selectively inhibits the current of the voltage-gated potassium channel Kv1.2 by occluding the ion conduction pathway. Here using molecular dynamics simulation as a docking method, the binding modes of MTx to three closely related channels (Kv1.1, Kv1.2 and Kv1.3) are examined. We show that MTx forms more favorable electrostatic interactions with the outer vestibule of Kv1.2 compared to Kv1.1 and Kv1.3, consistent with the selectivity of MTx for Kv1.2 over Kv1.1 and Kv1.3 observed experimentally. One salt bridge in the bound complex of MTx-Kv1.2 forms and breaks in a simulation period of 20****ns, suggesting the dynamic nature of toxin-channel interactions. The toxin selectivity likely arises from the differences in the shape of the channel outer vestibule, giving rise to distinct orientations of MTx on block. Potential of mean force calculations show that MTx blocks Kv1.1, Kv1.2 and Kv1.3 with an IC_50_ value of 6 µM, 0.6****nM and 18 µM, respectively.

## Introduction

Various short peptides isolated from venoms of poisonous creatures, such as scorpions, bees, snakes, cone snails, and sea anemones, inhibit K^+^ and other cationic channels by physically occluding the ion conduction pathway. The backbone of these peptide toxins is stabilized by several disulfide bridges [1]. The rigid backbone of these toxins is believed to be important for their abilities to inhibit K^+^ channels with high affinities. The secondary structure of the toxins is frequently characterized by an α-helix and a double- or triple-strand anti-parallel β-sheet. These toxins have found various potential applications in pharmaceutics as well as physiological studies of ion channels. For example, several mutants of the toxin ShK isolated from the sea anemone have been developed as selective blockers of the voltage-gated K^+^ channel Kv1.3, which is a target for immunosuppression [2]. The synthetic form of the ω-conotoxin MVIIA, which is a voltage-gated calcium channel blocker, has been approved to treat severe pain [3].

**Figure 1:**
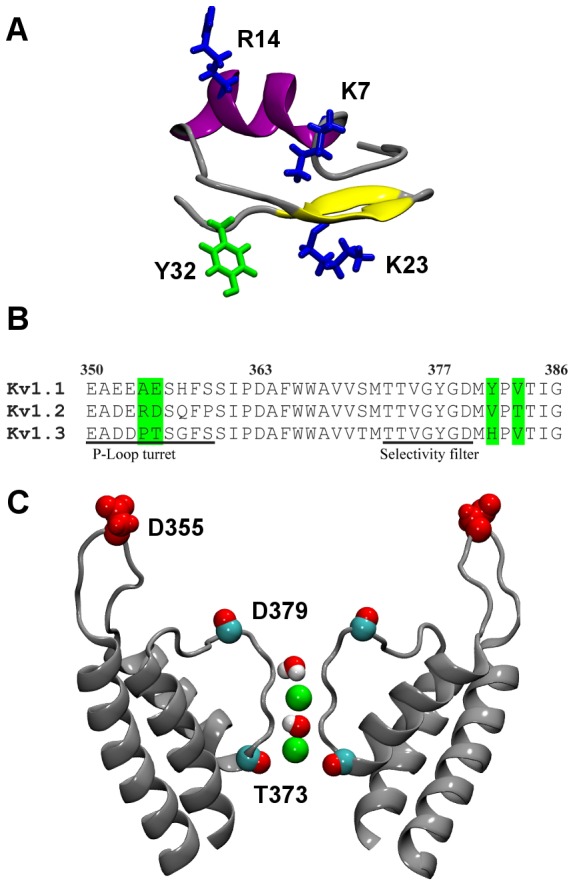
Structure of MTx and Kv1.2. (A) The secondary structure of MTx (PDB ID 1TXM [32]). α-Helix is shown in purple and β-sheets in yellow. The side chains of four key residues are highlighted. (B) Sequence alignment of the pore domains of Kv1.1, Kv1.2 and Kv1.3. Key different residues are highlighted in green. The P-loop turret and selectivity filter regions are indicated with horizontal bars. Numbering is that of Kv1.2. (C) Pore domain of Kv1.2 (PDB ID 3LUT [30]) viewed perpendicular to the channel axis. Two channel subunits are shown. The side chain of Asp355 and the backbone carbonyl groups of Thr373 and Asp379 are highlighted. Green spheres represent the two K^+^ ions in the selectivity filter. The two water molecules in the selectivity filter are also shown.

Maurotoxin (MTx) is a 34-residue peptide (VSCTG SKDCY APCRK QTGCP NAKCI NKSCK CYGC) isolated from the venom of *Scorpio maurus* [4]. The C-terminus of MTx is amidated and thus does not carry a negative charge at neutral pH. Figure 1A shows that the secondary structure of MTx contains an α-helix and two anti-parallel β-sheets. MTx has been shown to inhibit one subtype of voltage-gated K^+^ channels of the *Shaker* family (Kv1.2) and calcium-activated K^+^ channels of intermediate-conductance (IK_Ca_) with nanomolar affinities [4], [5], [6]. MTx is special in that its backbone is interconnected by four disulfide bridges (Cys3-Cys24, Cys9-Cys29, Cys13-Cys19 and Cys31-Cys34), rather than three disulfide bridges commonly found in other Kv1 channel toxin blockers. MTx has a particular high affinity for Kv1.2 (IC_50_ = 0.8****nM), whereas its affinities for Kv1.1 (IC_50_ = 37****nM or >100) and Kv1.3 (IC_50_ = 150****nM or 3 µM) are significantly lower [4], [5], [7]. Here, two IC_50_ values measured from channels expressed in different cell lines are quoted for Kv1.1 and Kv1.3 (more details will be described below). This is in contrast to many other Kv1 channel blockers such as charybdotoxin (ChTx) [8], ShK [9] and OSK1 [10], which are more effective for Kv1.3 or Kv1.1 than Kv1.2. MTx shows high selectivity for Kv1.2 over Kv1.1 and Kv1.3, although these channels differ in only several positions at the P-loop turret and near the selectivity filter (Figure 1B). A small ring of four aspartate residues at position 379 is located just above the selectivity filter of Kv1.2, whereas a larger acidic ring at position 355 of the P-loop turret is located about 10 Å above it (Figure 1C).

Due to the unique selectivity profile of MTx for Kv1.2 and IK_Ca_, a number of experimental [5], [6], [7], [11], [12], [13], [14], [15] as well as theoretical [16], [17], [18] studies have been carried out to understand the binding modes of MTx to K^+^ channels. These studies are consistent with Lys23 of MTx being the key residue which protrudes into the selectivity filter of Kv1.2 on binding. The mechanism of block by MTx has been believed to be similar to other peptide blockers such as ChTx which carries three disulfide bridges [11]. However, how MTx interacts with the outer vestibular wall of Kv1.2 and other channels has not been resolved. For example, Fu et al. [16] found that Lys30 of MTx is a key residue coupled with Asp379 of Kv1.2, whereas Yi et al. [17] suggested that Lys7 of MTx is the residue in contact with Asp379. Yet, Visan et al. [5] believe that Lys7 of MTx should be in close proximity to Asp363 of Kv1.2.

Experimental studies performed on different types of cells expressing Kv1.1-Kv1.3 channels have demonstrated that MTx is selective for Kv1.2 over Kv1.1 and Kv1.3 [4], [5], [7]. However, half-maximal inhibitory concentrations (IC_50_) of MTx for Kv1.1 and Kv1.3, determined experimentally, appear to vary enormously. For example, experiments performed on *Xenopus* oocytes showed that MTx inhibited the current of Kv1.1 with an IC_50_ of 37****nM and Kv1.3 with an IC_50_ of 150****nM [4], whereas it did not inhibit Kv1.1 and Kv1.3 expressed in Chinese hamster ovary cells at a concentration of 100****nM [7]. In contrast, Visan et al. [5] reported an IC_50_ value of 3 µM for the inhibition of Kv1.3 by MTx. Thus, the binding affinities of MTx for Kv1.1 and Kv1.3 appeared to be sensitive to experimental conditions. The different binding affinities of MTx reported experimentally need to be verified.

Here, using molecular dynamics (MD) simulations with distance restraints as a docking method, the binding modes of MTx to Kv1.1, Kv1.2 and Kv1.3 are examined. The bound complexes predicted show that MTx forms less a hydrogen bond on binding to Kv1.1 and Kv1.3 than to Kv1.2. In the MTx-Kv1.2 complex, three key interacting residue pairs (Lys23-Tyr377, Arg14-Asp355 and Lys7-Asp363) are identified, consistent with mutagenesis experiments of Visan et al. [5]. The IC_50_ values for the unbinding of MTx from the complexes are derived with potential of mean force calculations, which suggest that MTx inhibits Kv1.1 and Kv1.3 with micromolar affinities. The selectivity of MTx for Kv1.2 over Kv1.1 and Kv1.3 likely arises from the steric effects by residue 381 near the selectivity filter.

## Computational Methods

### Molecular Dynamics as a Docking Method

Different methods including rigid-body molecular docking [18], [19], [20], molecular docking with limited flexibility [21], [22], Brownian dynamics simulation [23], [24], [25], and MD simulation with distance restraints (biased MD) [26], have been used to predict the binding modes between various toxins and channels. In molecular docking methods and Brownian dynamics simulation, the flexibility of proteins and the entropy of water are ignored. In contrast, both protein flexibility and water entropy are taken into account in biased MD. However, biased MD requires at least one toxin-channel interaction residue pair to be identified from experimental data at the beginning of simulations. In biased MD, a harmonic potential is applied to maintain the distance between one or several predefined residue pairs below one or several predefined upper boundaries.

For MTx, both theoretical [16], [17] and experimental [5], [7] studies are consistent with Lys23 occluding the selectivity filter of Kv1.2. In addition, Lys23 is located in the β-strand immediately preceded by the α-helix (Figure 1A), corresponding to the position of the key lysine residue found in closely related toxins [27], [28]. Thus, an interaction residue pair between Lys23 of MTx and Tyr377 in the selectivity filter of Kv1.2 is identified. The available knowledge on the Lys23-Tyr377 residue pair enables us to use biased MD to predict the position of MTx relative to the outer vestibule of Kv1.1, Kv1.2 and Kv1.3 on block.

### Molecular Dynamics Simulations

We construct homology models of the pore domains of human Kv1.1 and Kv1.3 channels on the crystal structure of rat Kv1.2 [29], [30] using the methods detailed elsewhere [20], [31]. These three channels share >90% sequence identity in the pore domain. Human and rat Kv1.2 channels differ only at position 355 in the pore domain, where it is a glutamate in rat and an aspartate in human. Each channel is then embedded in a 2-oleoyl-1-palmitoyl-*sn*-glycero-3-phosphocholine bilayer (∼80 lipids/leaflet) and a box of explicit water (∼15,000 molecules). Approximately 58 K^+^ and Cl^-^ ions each are added into each system, corresponding to a salt concentration of 0.2 M. Two K^+^ ions are moved to the S2 and S4 binding sites of the selectivity filter, with the S1 and S3 ion binding sites occupied by two water molecules (Figure 1C). Each system is equilibrated for 5****ns, and the sizes of the simulation boxes are ∼85×85×105 Å^3^ after the 5-ns simulation. In our previous work [31], we have shown that the P-loop turrets of Kv1.1 and Kv1.2 move outward substantially in the presence of the voltage-sensing domain. Thus, to model the P-loop turret correctly in the reduced channel model in which the voltage-sensing domain is truncated, the centers of mass of the residues at position 355 in the P-loop turrets of Kv1.1 and Kv1.2 (Figure 1B) are maintained to be at least 19–20 Å from the channel central axis.

After the 5-ns equilibration, we place MTx (PDB ID 1TXM [32]) into each simulation box, ∼15 Å above the position where the toxin is fully bound. A flat-bottom harmonic distance restraint is applied between the side chain nitrogen atom of MTx-Lys23 and the carbonyl group of the channel residue Gly376. When Lys23 protrudes into the selectivity filter and forms hydrogen bonds with Tyr377, the side-chain nitrogen is ∼4 Å above the plane of the carbonyl groups of Gly376. Thus, the upper boundary of the distance between Lys23-Gly376 is progressively reduced from 15 Å to 3 Å over a simulation period of 5****ns, so that Lys23 of MTx is gradually pulled into the selectivity filter. The force constant of the distance restraint is set to 1 kcal/mol/Å^2^. The backbone of MTx is maintained rigid, so that no significant conformational changes in MTx are induced by the distance restraint. The simulation is extended for 15****ns with the distance restraint removed, allowing the toxin-channel complexes to evolve to energetically favorable states. Each simulation is repeated a second time with different random initial velocities, and a third time with a different toxin orientation. The two different toxin orientations for Kv1.2 are shown in Figure S1 of the Supporting Information. Multiple simulations starting from different random initial velocities have been widely used to enhance the ability of MD to probe the dynamics of biomolecules such as proteins [33], [34]. Consistent binding modes by MTx are observed in the three simulations in all cases.

All molecular dynamics simulations are performed under periodic boundary conditions using NAMD [35] version 2.8. The CHARMM36 force field for lipids [36] and proteins [37] and the TIP3P model [38] for water are used to describe the interatomic interactions in the system. The switch and cutoff distances for short-range interactions are set to 8.0 Å and 12.0 Å, respectively. The long-range electrostatic interactions are accounted for using the particle mesh Ewald method (grid spacing ≤1.0 Å). Bond lengths are maintained rigid with the SHAKE [39] and SETTLE [40] algorithms, allowing a time step of 2 fs to be used. The temperature is maintained constant at an average value of 300 K by using the Langevin dynamics (damping coefficient 1 ps^–1^). Nosé-Hoover Langevin Piston method [41] is used to maintain a constant pressure of 1 atm. The barostat oscillation and damping time scale are set to 200 ps and 100 ps, respectively. The pressure coupling is semiisotropic. Trajectories are saved every 20 ps for analysis.

### Umbrella Sampling

We derive with umbrella sampling the potential of mean force (PMF) profile for the unbinding of MTx from each channel along the channel axis. Based on the PMF profile, the IC_50_ for the toxin block can be calculated [42]. We use steered molecular dynamics to pull the toxin out from the binding site, and generate the starting structures of the umbrella windows spaced at 0.5 Å intervals. The toxin backbone is maintained rigid during the pulling, whereas the backbone atoms of the channel are fixed.

The center of mass (COM) of the toxin backbone is restrained to the center of each umbrella window using a harmonic force constant of 20–40 kcal/mol/Å^2^. The COM of the channel is at *z* = 0 Å. The COM of the toxin backbone is maintained in a cylinder of 8 Å in radius centered on the channel axis, using a flat-bottom harmonic restraint. The radius of the cylinder is chosen such that the restraining potential is always zero when the toxin is bound, and only occasionally non-zero when the toxin is in the bulk. This allows all the degrees of freedom of the toxin to be adequately sampled without bias. Each umbrella window is simulated for at least 5****ns until the depth of the PMF profile changes by <0.5 *kT* over the last 1****ns. The first 1****ns of each window is removed from data analysis. The *z* coordinate of the toxin COM is saved every 1 ps for analysis. The weighted histogram analysis method is used to construct the PMF profile [43]. The IC_50_ value is derived using the following equation [20], [42]:
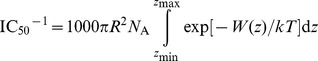
(1)where *R* is the radius of the cylinder (8 Å), *N*_A_ is Avogadro's number, *z*_min_ and *z*_max_ are the boundaries of the binding site along the reaction coordinate (*z*), *W(z)* is the PMF, and *kT* assumes the usual significance. We note here that Equation 1, which is derived rigorously from first principles [42], is valid only when appropriate flat-bottom cylindrical restraints are applied when deriving the profile of PMF.

## Results and Discussion

### Binding to Kv1.2

MTx inhibits the current of Kv1.2 potently with an IC_50_ of 0.7–0.8****nM [4], [5], [7]. The binding modes of MTx to Kv1.2 have been suggested to be similar to that of ChTx [11]. Here, using MD as a docking method, the binding mode between MTx and Kv1.2 is predicted. The bound complex of MTx-Kv1.2 shows that while Lys23 of MTx occludes the ion conduction conduit, Lys7 and Arg14 form two salt bridges with Asp363 and Asp355 of Kv1.2, respectively.

**Figure 2:**
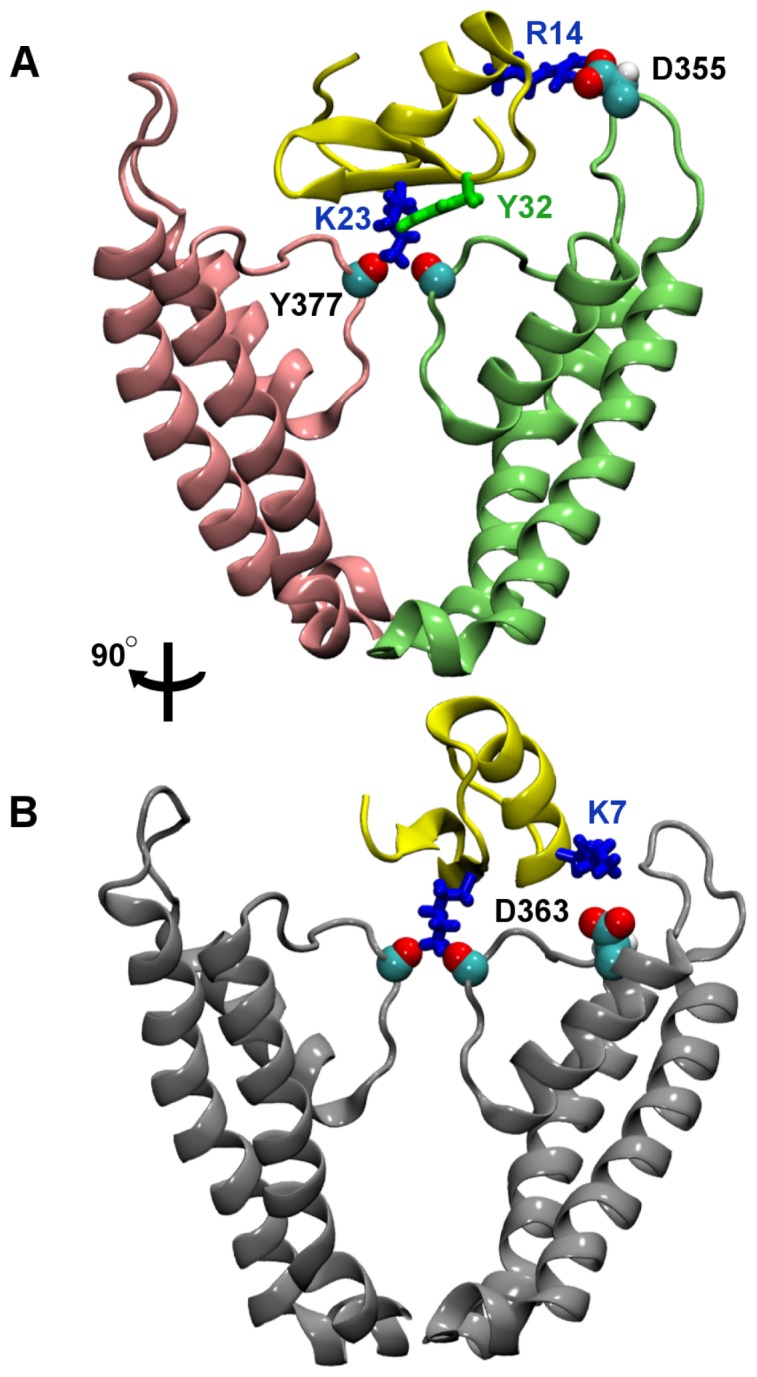
MTx bound to Kv1.2. In (A), two key residue pairs Lys23-Tyr377 and Arg14-Asp355 are highlighted. Two channel subunits are shown for clarity. (B) The MTx-Kv1.2 complex rotated by approximately 90° clockwise from that of (A). The third key residue pair Lys7-Asp363 is highlighted in (B).

**Figure 3:**
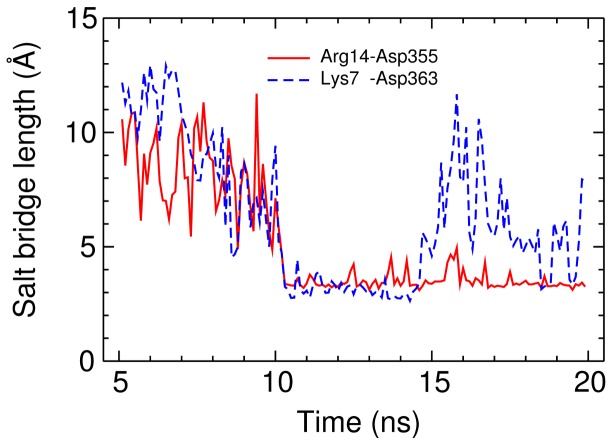
Time evolution of the salt bridge lengths. The lengths of the salt bridges Arg14-Asp355 and Lys7-Asp363 formed in the MTx-Kv1.2 complexes as a function of the simulation time over the last 15****ns.

To predict the bound complex of MTx-Kv1.2, we apply a distance restraint between Lys23 of MTx and Gly376 of Kv1.2. A harmonic force is applied if the distance between the side-chain nitrogen of Lys23 and the carbonyl group of Gly376 is above the upper boundary of the distance restraint. Otherwise, the force is zero if the distance between Lys23-Gly376 is lower than the upper boundary. In the presence of the distance restraint, the toxin is gradually drawn to the outer vestibule of Kv1.2. Figure 2A displays a representative configuration showing the position of MTx relative to the outer vestibule of Kv1.2 after the docking simulation totaling 20****ns. Two key contacts of the MTx-Kv1.2 complex are shown. One firm contact is inside the selectivity filter, where Lys23 of MTx forms hydrogen bonds with the carbonyl groups of Tyr377 from the four channel subunits (Figure 2A). A hydrogen bond is considered to be formed if the donor and acceptor atoms (nitrogen or oxygen) are within 3 Å of each other and the donor-hydrogen-acceptor angle is ≥150° [44]. The other contact is between Arg14 of MTx and Asp355 on the P-loop turret of Kv1.2, where these two residues form a hydrogen bond and salt bridge (Figure 2A). A salt bridge is considered to be formed if the distance is less than 4 Å between a side chain oxygen atom from an acidic residue and a nitrogen atom from a basic residue [45]. Figure 2B shows that Lys7 of MTx forms the third strong contact with Asp363 on the outer vestibular wall of Kv1.2. The Lys7-Asp363 appears to be less stable than Arg14-Asp355; Lys7 occasionally forms a hydrogen bond with Gln357 in the P-loop turret. Figure 3 shows the lengths of the salt bridges Arg14-Asp355 and Lys7-Asp363 as a function of the simulation time over the last 15****ns. The Lys7-Asp363 salt bridge forms at 10****ns but breaks at 15****ns, whereas the Arg14-Asp355 salt bridge remains stable between 10 and 20****ns. In the second and third docking simulations, the two salt bridges were also observed to form and break. Thus, the simulations show that the interactions between the MTx and the outer vestibule are highly dynamic, although Lys23 persistently occludes the ion conduction pathway. Similar dynamic toxin-channel interactions have been observed in previous simulations of ChTx and Kv1.3 [20].

Mutagenesis experiments and double mutant cycle analysis have suggested the strong coupling between the Tyr32 of MTx and Val381 of Kv1.2 [5]. Consistent with these experimental observations, our binding mode shows that Tyr32 interacts intimately with residues near the entrance of the selectivity filter (Figure 2A). The minimum inter-residue distance of Tyr32-Val381 is 2.7±1.1 Å on average, indicating the strong coupling of this residue pair.

Double mutant cycle analysis has also suggested that Arg14 may be coupled with Asp355 [5]. Our model displayed in Figure 2 is consistent with mutagenesis experiments [5], which suggest that Arg14 is coupled with Asp355, and Lys7 is coupled with Asp363. We note that two acidic residues Asp352 and Glu353 are in close proximity to Asp355. These two residues could form salt bridges with MTx if Asp355 is mutated to a neutral or basic amino acid. This would explain the minimal effect on MTx binding affinity caused by the alanine mutation of Asp355 observed experimentally [5]. Thus, our model of MTx-Kv1.2 is in accord with the experimental measurements of Visan et al. [5].

To further verify the binding mode of MTx-Kv1.2 predicted by the docking using biased MD simulation, molecular docking calculations using the rigid-body docking program ZDOCK [46], [47] are performed. This docking program has been applied to numerous similar toxin-channel systems [17], [18], [19], [20], [48]. We assume that the docking pose generated by the program is correct if Lys23 protrudes into the selectivity filter. If the same MTx structure as that used in biased MD were used in ZDOCK, the correct docking pose obtained is found to be nearly identical to that predicted from biased MD. Therefore, we select a different structure of MTx, namely, the 21^st^ NMR structure in 1TXM [32], and submit this structure to ZDOCK. The top-ranked correct docking pose is then equilibrated for 10****ns using MD without restraints. The MTx-Kv1.2 complex after the 10-ns equilibration is shown in Figure S2 of the Supporting Information. The MTx-Kv1.2 complex predicted by ZDOCK is virtually identical to that shown in Figure 2, indicating that the MTx-Kv1.2 complex obtained from biased MD is reliable.

### Binding to Kv1.1 and Kv1.3

It has been demonstrated that MTx inhibits Kv1.1 and Kv1.3 with much lower affinities than Kv1.2 [4], [5], [7]. In accord with experiment, we show that less favorable interactions are formed in the MTx-Kv1.1 and MTx-Kv1.3 complexes than in MTx-Kv1.2. Only two stable hydrogen bonds are observed in MTx-Kv1.1 and MTx-Kv1.3, whereas three hydrogen bonds are observed in MTx-Kv1.2.

**Figure 4:**
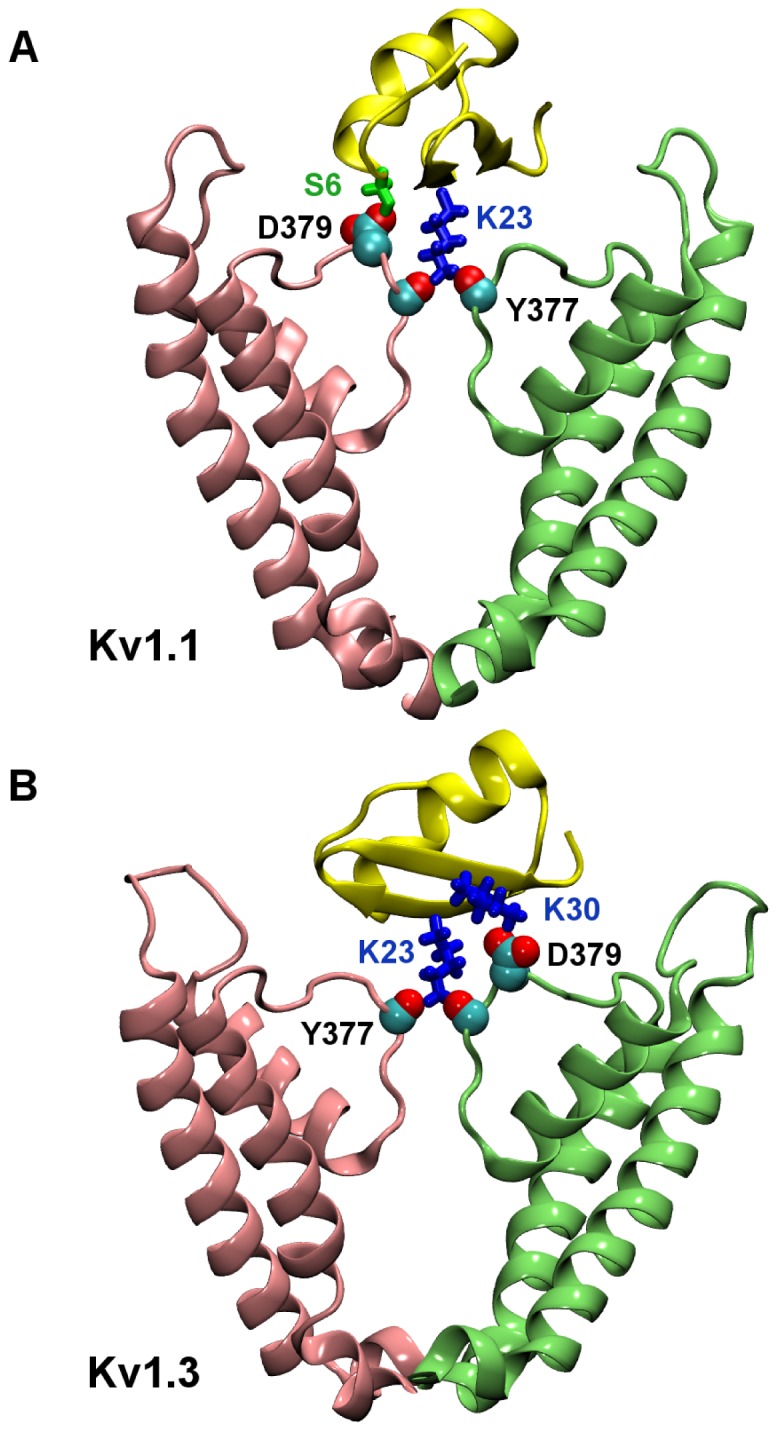
MTx bound to Kv1.1 and Kv1.3. In (A), two key residue pairs Lys23-Tyr377 and S6-Asp379 are highlighted. In (B), residue pairs Lys23-Tyr377 and Lys30-Asp379 are highlighted.

Figure 4A shows the position of MTx relative to the outer vestibular wall of Kv1.1 after 20-ns docking calculations. The two residue pairs (Lys23-Tyr377 and Ser6-Asp379) forming hydrogen bonds are highlighted in the figure. In the MTx-Kv1.1 complex, Lys23 of MTx wedges into the selectivity filter of Kv1.1, as assumed at the start of the docking simulation. The electrostatic interactions between Lys23 and the selectivity filter are strong, owing to the negative electrostatic field in the selectivity filer and the hydrogen bond formed between Lys23-Tyr377. A second hydrogen bond is observed between the residue pair Ser6-Asp379. The MTx-Kv1.1 is stable in the simulation over the last 15****ns, likely due to these two hydrogen bonds as well as hydrophobic interactions and other transient hydrogen bonds.

**Figure 5:**
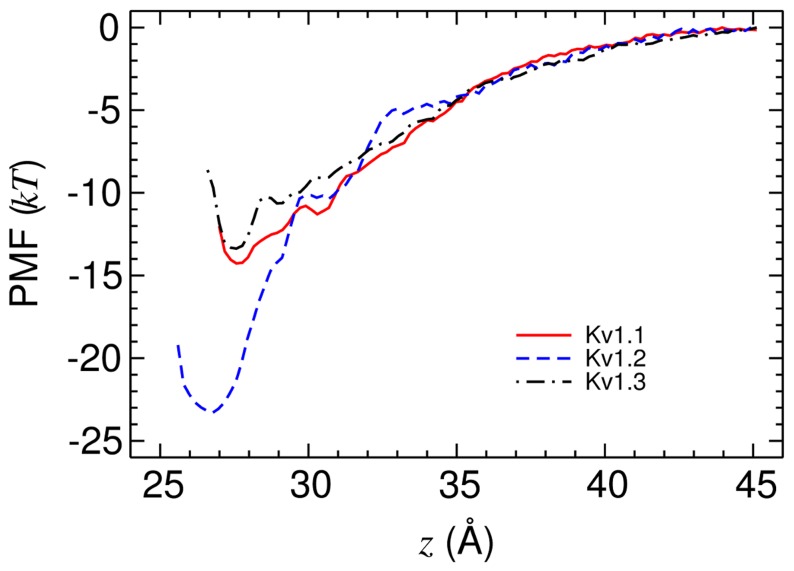
The PMF profiles for the unbinding of MTx from Kv1.1, Kv1.2 and Kv1.3. The reaction coordinate is the distance between the centers of mass of the channel and toxin along the channel axis (*z*).

Figure 4B shows the position of MTx relative to the outer vestibular wall of Kv1.3 predicted from MD docking. Similar to MTx-Kv1.1, a second hydrogen bond on the outer vestibule is observed in addition to Lys23-Tyr377. However, the second hydrogen bond in the MTx-Kv1.3 is formed by the residue pair Lys30-Asp379 rather than S6-Asp379 in the MTx-Kv1.1 complex. The MTx-Kv1.3 complex is also stable over the last 15****ns of simulation. The less favorable interactions by MTx-Kv1.1 and MTx-Kv1.3 are reflected in their shallower PMF profiles and higher IC_50_ values (see Figure 5).

### Binding Energetics and Affinities

To verify the binding modes predicted, we construct the PMF profiles for the unbinding of the complexes and derive the corresponding IC_50_ values of toxin inhibition. The PMF profiles for the unbinding of MTx from Kv1.1, Kv1.2 and Kv1.3 along the channel axis are constructed using the umbrella sampling technique.

The converged PMF profiles are displayed in Figure 5. The shape of the PMF profiles is similar in the region between *z* = 35 Å and *z* = 45 Å. The depths of the PMF profiles for both Kv1.1 and Kv1.3 are approximately −14 *kT*, corresponding to an IC_50_ value of 6 and 18 µM, respectively. However, the PMF profile for Kv1.2 is observed to be significantly deeper. The depth of the Kv1.2 PMF profile is about −23 *kT*, corresponding to an IC_50_ value of 0.6****nM, which is comparable to the experimental value of 0.7–0.8****nM [4], [5]. Thus, the calculations of PMF demonstrate that MTx is selective for Kv1.2 over Kv1.1 and Kv1.3, consistent with experiment [4], [5], [7]. The interacting residue pairs between MTx and the three channels identified from the umbrella sampling simulation of the window at the minimum PMF are displayed in Table S1 of the Supporting Information.

One of the efficient empirical methods to calculate the binding free energy of ligand is the linear interaction energy (LIE) approximation [49]. In the LIE method, the binding free energy Δ*G*_bind_ can be expressed as [49]:
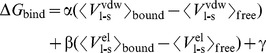
(2)where the subscript l-s denotes ligand-surrounding interactions, α, β and γ are constants that can be fitted to experimental Δ*G*_bind_ values. Surrounding denotes all parts of the system except the ligand. *V* ^vdw^ and *V* ^el^ represent the van der Waals and electrostatic interaction energies, respectively. Table 1 shows the energy terms of Equation 2 for the binding of MTx to Kv1.1-Kv1.3 channels. Two conclusions can be drawn. First, the variation of the interaction energies as a function of time is large, which would give rise to large inherent errors in the Δ*G*_bind_ values predicted by Equation 2. For example, the standard deviations of the electrostatic interaction energies are as large as 70 kcal/mol (∼120 *kT*). In contrast, the uncertainty of the depths of the PMF profiles displayed in Figure 5 is only about 0.4 *kT*. Second, the interaction energies appear not to be correlated with the binding free energy, indicating that the entropic component of Δ*G*_bind_, which is ignored in Equation 2, is critical for an accurate prediction of Δ*G*_bind_.

**Table 1:** The average changes in van der Waals (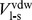) and electrostatic (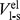) interaction energies (kcal/mol) between MTx and the surroundings due to the binding of MTx to Kv1.1–Kv1.3 channels.

Channel	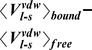	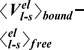	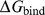 (*kT*)
Kv1.1	−51±11	−416±55	−12.0
Kv1.2	−22±12	−17±76	−21.2
Kv1.3	−43±12	−265±67	−10.9

Standard deviations are shown. Δ*G*_bind_ is calculated as 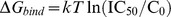, where C_0_ is 1 M. The IC_50_ values are 6 µM, 0.6****nM and 18 µM for Kv1.1, Kv1.2 and Kv1.3, respectively.

### Structural Basis of Selectivity

MTx has been shown to be selective for Kv1.2 over Kv1.1 and Kv1.3 at nanomolar toxin concentrations [4], [5], [7]. These three channels are >90% identical in their pore domains, and differ only at several positions in the P-loop turret and near the selectivity filter (Figure 1B). The residue at position 381 near the selectivity filter is believed to largely determine the selectivity of MTx for Kv1.2 over Kv1.3 [5]. The modes for MTx bound to the three channels suggest that the selectivity of MTx arises from the steric effects caused by the residue 381 of the channel. The hydrophobic interaction between the toxin and the residue 381 of the channel likely contributes a rather secondary effect, because valine (Kv1.2) is similar to tyrosine (Kv1.1) in hydrophobicity, as suggested by recent theoretical calculations [50].

**Figure 6:**
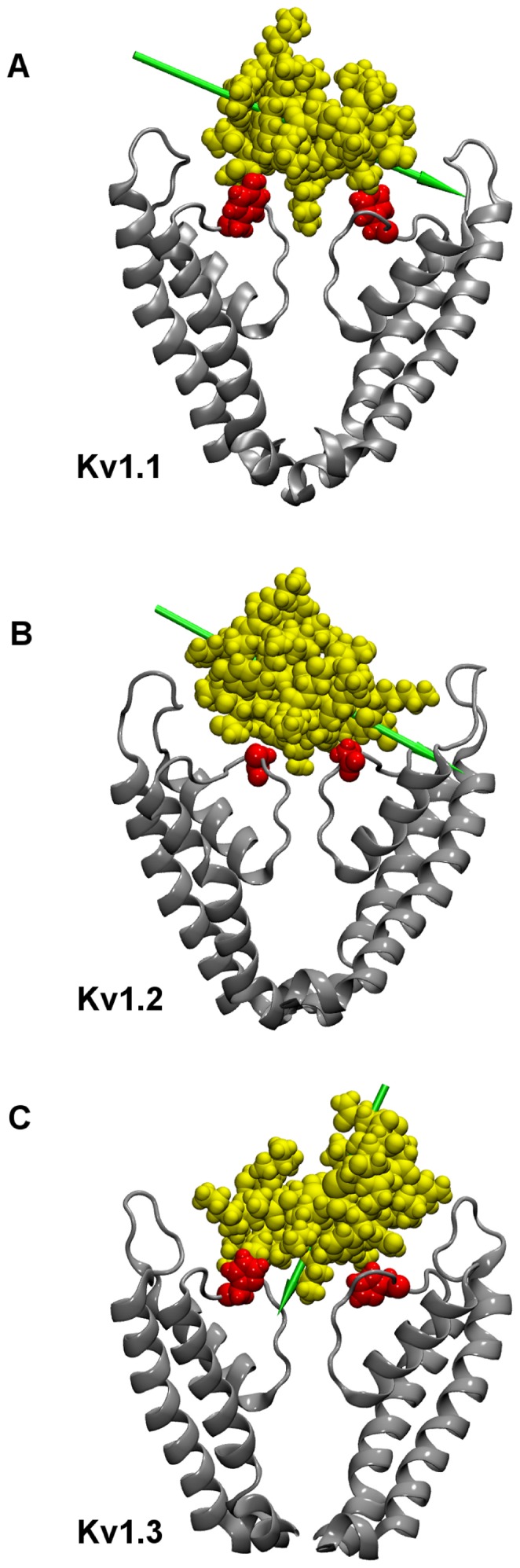
The position of MTx (yellow) relative to Kv1.1-Kv1.3 channels. The key residue 381 is highlighted in red. Green arrows represent the dipole moment of MTx.

Figure 6 demonstrates that the orientations of MTx on binding to Kv1.1-Kv1.3 are significantly different. For example, the dipole moment of MTx fluctuates on an average of approximately 45°, 60° and 20° with respect to the channel axis when the toxin is bound to Kv1.1, Kv1.2 and Kv1.3, respectively. The distinct binding orientations must be related to the residues at position 381 of the channel (Figure 1B). For example, the residues Tyr381 in Kv1.1 and His381 in Kv1.3 are bulkier than the residue Val381 in Kv1.2. As a result, MTx binds closer to Kv1.2 than to Kv1.1 and Kv1.3, as illustrated in Figure 6. At the bound state, the COM of MTx is 27 Å from the COM of Kv1.2, whereas the COM of MTx is 28 Å from the COM of Kv1.1 and Kv1.3 (Figure 5). The differences in the size of the residue at position 381 may lead to different shapes on the channel surface, such that the outer vestibule of Kv1.2 provides a better receptor site for MTx.

If the channel residue at position 381 were critical for toxin selectivity, one would expect that MTx should form similar salt bridges with the outer vestibular wall of Kv1.2 and H381V mutant Kv1.3. Following this hypothesis, computational mutagenesis calculations are carried out. Specifically, His381 of Kv1.3 in the MTx-Kv1.3 complex is mutated to valine, corresponding to the residue at position 381 in Kv1.2. The new complex is equilibrated for 10****ns using MD without restraints. The MTx-[H381V] Kv1.3 complex after the equilibration is displayed in Figure S3. A new salt bridge, Arg14-Asp353, not found in the MTx-Kv1.3 complex, is formed. This salt bridge can be considered as equivalent to the Arg14-Asp355 salt-bridge in the MTx-Kv1.2 complex, In addition, Lys7 of MTx is observed to be in close proximity to Asp363 of the mutant Kv1.3, with the average minimum distance being <6 Å, consistent with the Lys7-Asp363 salt bridge in the MTx-Kv1.2 complex. Our computational mutagenesis calculations support the critical role of residue 381 in MTx selectivity.

### Conclusions

The bound complexes between the scorpion toxin MTx and three voltage-gated potassium channels of the *Shaker* family (Kv1.1-Kv1.3) are predicted using MD simulation as a docking method. The MTx-Kv1.2 complex reveals that the side chain of Lys23 firmly occludes the ion conduction conduit of the channel by forming strong electrostatic interactions with the channel selectivity filter (Figure 2). At the same time, MTx forms two additional hydrogen bonds with residues on the outer vestibular wall of Kv1.2. One hydrogen bond (Arg14-Asp355) appears to be stable after its formation at 10****ns, while the second hydrogen bond (Lys7-Asp363) is observed to be unstable and subsequently breaks at 15****ns (Figure 3). This highlights the dynamic nature of toxin-channel interactions. Our model of MTx-Kv1.2 is in agreement with mutagenesis experiments [5]. In the computational model proposed by Yi et al. [17], Lys7 of MTx forms a salt bridge with Asp379, whereas in our model Lys7 is in closer proximity to Asp363.

The complexes MTx-Kv1.1 and MTx-Kv1.3 show that two stable hydrogen bonds are formed in both cases, including one inside and the other just outside the selectivity filter (Figure 4). These two hydrogen bonds are sufficient for stabilizing the toxin-channel complex. The PMF profiles constructed show that the binding affinities of MTx to Kv1.1 (IC_50_ = 6 µM) and Kv1.3 (IC_50_ = 18 µM) are in the micromolar range. Thus, our calculations indicate that MTx is capable of inhibiting Kv1.1 and Kv1.3, albeit it inhibits Kv1.2 at a four orders of magnitude lower concentration.

In conclusion, structural models for MTx bound to Kv1.1, Kv1.2 and Kv1.3 channels are generated using MD simulation as a docking method. Such a docking method may be applied to other toxin-channel systems to rapidly predict the binding modes. Our models of MTx-Kv1.1, MTx-Kv1.2 and MTx-Kv1.3 can explain the selectivity of MTx for Kv1.2 over Kv1.1 and Kv1.3 observed experimentally, and suggest that toxin selectivity arises from the steric effects by residue 381 near the channel selectivity filter.

## Supporting Information

Figure S1 **The two distinct positions of MTx relative to Kv1.2 at the start of the MD docking simulations.** The toxin backbones are shown in green and blue, and channel backbone in silver. Only two of the four channel subunits are shown for clarity.
(TIFF)

Figure S2 **MTx bound to Kv1.2 predicted from ZDOCK and a 10-ns unbiased MD simulation.** In (A), two key residue pairs Lys23-Tyr377 and Arg14-Asp355 are highlighted. Two channel subunits are shown for clarity. (B) The MTx-Kv1.2 complex rotated by approximately 90° clockwise from that of (A). The third key residue pair Lys7-Asp363 is highlighted in (B).
(TIFF)

Figure S3 **MTx bound to H381V mutant Kv1.3 after 10 ns of MD simulation.** Two interacting residue pairs, Arg14-Asp353 and Lys7-Asp363, are indicated. Two of the channel subunits are highlighted in pink and lime, respectively. Toxin backbone is shown as yellow ribbons.
(TIFF)

Table S1 **Interacting residue pairs between MTx and the three channels, Kv1.1-Kv1.3.** The 5-ns umbrella sampling simulation of the window at the minimum PMF is used for analysis. The minimum distances (Å) of each residue pair averaged over the last 4****ns are given in the brackets, together with standard deviations.
(DOC)
