# Supplementary figures and images for: Structural Basis of the Selective Block of Kv1.2 by Maurotoxin from Computer Simulations
Source: PLoS One. 2012 Oct 10;7(10):e47253. doi: 10.1371/journal.pone.0047253 (PMC3468451; doi:10.1371/journal.pone.0047253)

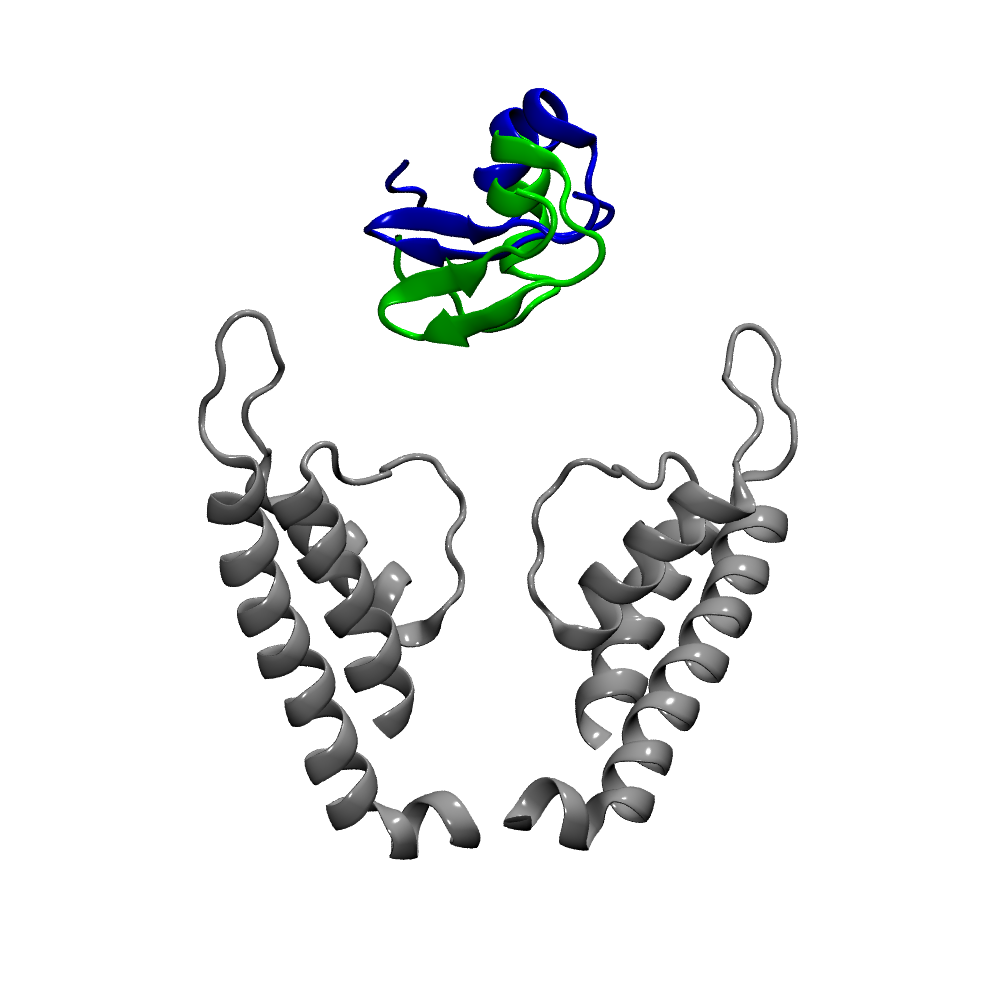

Supplement: Figure S1 — The two distinct positions of MTx relative to Kv1.2 at the start of the MD docking simulations. The toxin backbones are shown in green and blue, and channel backbone in silver. Only two of the four channel subunits are shown for clarity. (TIFF) [file pone.0047253.s001.tiff]

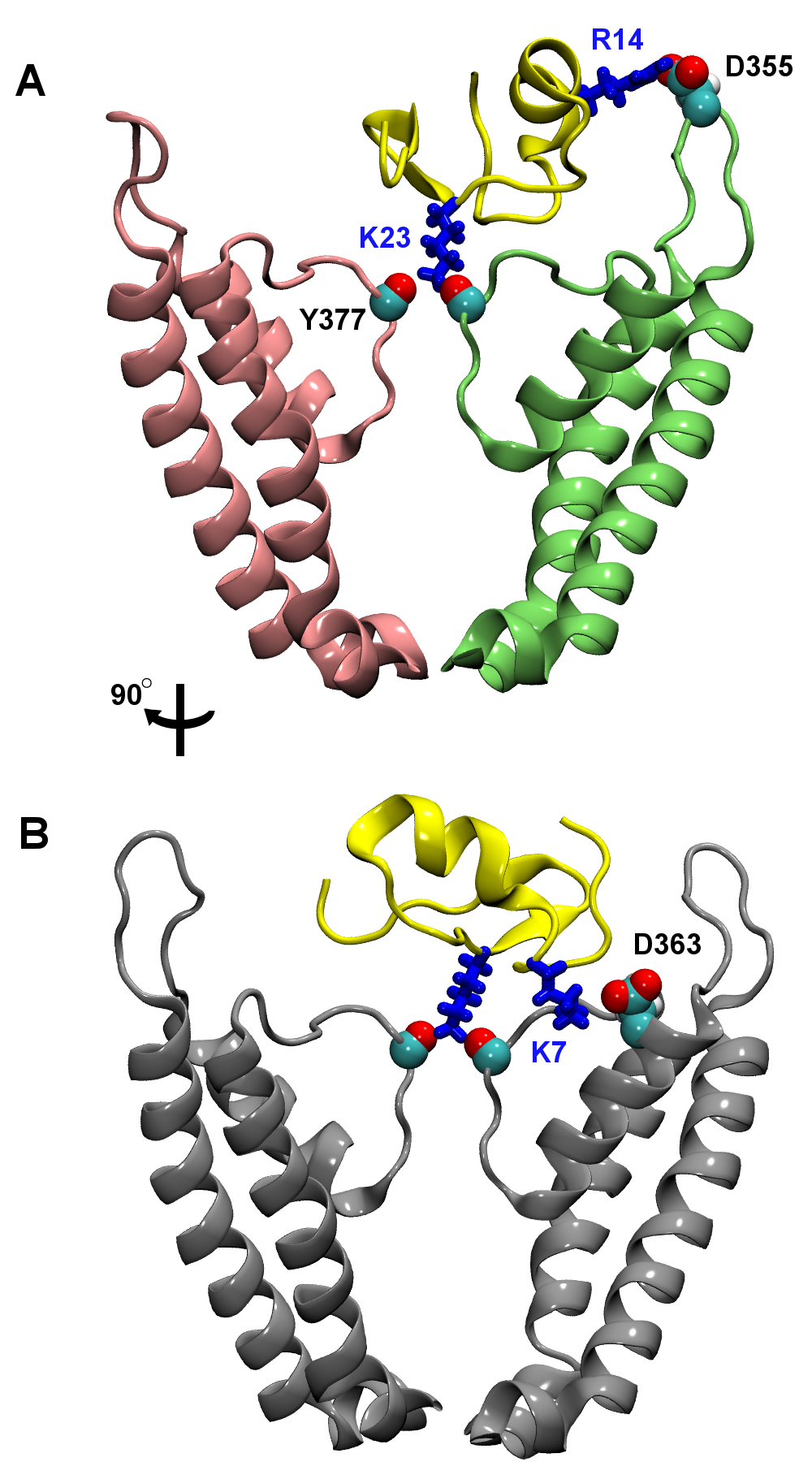

Supplement: Figure S2 — MTx bound to Kv1.2 predicted from ZDOCK and a 10-ns unbiased MD simulation. In (A), two key residue pairs Lys23-Tyr377 and Arg14-Asp355 are highlighted. Two channel subunits are shown for clarity. (B) The MTx-Kv1.2 complex rotated by approximately 90° clockwise from that of (A). The third key residue pair Lys7-Asp363 is highlighted in (B). (TIFF) [file pone.0047253.s002.tiff]

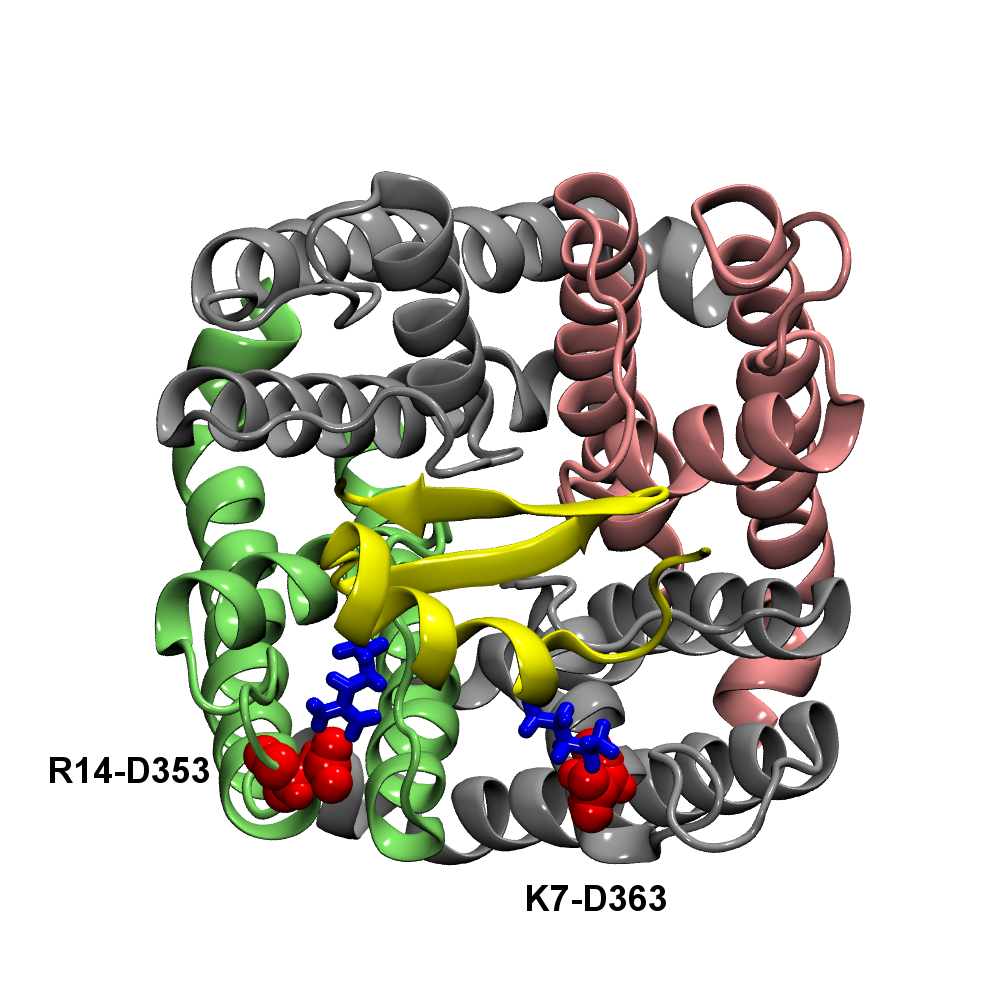

Supplement: Figure S3 — MTx bound to H381V mutant Kv1.3 after 10 ns of MD simulation. Two interacting residue pairs, Arg14-Asp353 and Lys7-Asp363, are indicated. Two of the channel subunits are highlighted in pink and lime, respectively. Toxin backbone is shown as yellow ribbons. (TIFF) [file pone.0047253.s003.tiff]
