# Supplementary material for: Structural Basis of the Selective Block of Kv1.2 by Maurotoxin from Computer Simulations
Source: PLoS One. 2012 Oct 10;7(10):e47253. doi: 10.1371/journal.pone.0047253 (PMC3468451; doi:10.1371/journal.pone.0047253)
Supplement: Table S1 — Interacting residue pairs between MTx and the three channels, Kv1.1-Kv1.3. The 5-ns umbrella sampling simulation of the window at the minimum PMF is used for analysis. The minimum distances (Å) of each residue pair averaged over the last 4ns are given in the brackets, together with standard deviations. (DOC) [file pone.0047253.s004.doc]

| MTx | Kv1.1 | Kv1.2 | Kv1.3 |
| --- | --- | --- | --- |
| S6 | D379 (2.8±1.2) | D379 (2.4±0.4) | D379 (3.0±0.4) |
| K7 | - | D363 (2.6±1.3) | T355 (1.9±0.3) |
| Y10 | Y381 (3.6±0.6) | D355 (4.0±0.6) | T355 (2.9±0.5) |
| R14 | - | D355 (1.8±0.3) | S356 (2.9±1.2) |
| K23 | Y377 (1.8±0.2) | Y377 (1.8±0.1) | Y377 (2.0±0.3) |
| K30 | Y381 (2.6±0.2) | V381 (3.9±0.5) | D379 (1.8±0.2) |
| Y32 | Y381 (2.5±0.3) | V381 (2.5±0.3) | D379 (1.8±0.2) |
